# An Overview of Hepatitis B Virus Surface Antigen Secretion Inhibitors

**DOI:** 10.3389/fmicb.2018.00662

**Published:** 2018-04-05

**Authors:** Alireza Mohebbi, Nazanin Lorestani, Alireza Tahamtan, Niki L. Kargar, Alijan Tabarraei

**Affiliations:** ^1^Student Research Committee, School of Medicine, Golestan University of Medical Sciences, Gorgan, Iran; ^2^Department of Microbiology, School of Medicine, Golestan University of Medical Sciences, Gorgan, Iran; ^3^Infectious Disease Research Center, Golestan University of Medical Sciences, Gorgan, Iran

**Keywords:** hepatitis B virus, chronic hepatitis B virus infection, HBsAg inhibitors, anti-viral natural products, RNA interference

## Abstract

Current anti-hepatitis B virus (HBV) regimen do not meet ideal result due to emerging resistance strains, cytotoxicity, and unfavorable adverse effects. In chronic HBV infection, high rates of sub-viral particles (SVPs) bearing HBV surface antigen (HBsAg) is a major obstacle regarding to raise effective immune responses and subsequently virus clearance. Development of potent HBsAg secretion inhibitors would provide a better insight into HBV immunopathogenesis and therapy. Investigating new non-toxic HBsAg secretion inhibitors targeting either viral or cellular factors could restore the immune response to remove virally infected hepatocytes after inhibiting SVPs. In this study, we overview several classes of HBV inhibitors with focus on their limitations and advantages over anti-HBsAg secretion potential.

## Introduction

Hepatitis B virus as a member of the *Hepadnaviridae* family is responsible for acute and chronic hepatitis in humans. While there are approved anti-viral drugs and vaccine, HBV infection remains as a main global public health challenge, which is reported to exist in more than 200 millions of people (Franco et al., 2012). HBV virions are small and composed of ∼3.2 kb partially double-stranded, relaxed circular DNA. The virus genome encodes four pre-genomic (serves as the template for genome synthesis), surface antigens (large, medium, and small), core (HBe and HBc), and trans-activator x (HBx) coding sequences.

In chronically infected individuals, the serum levels of HBsAg can be as high as 400 μg/mL with domination of non-infectious SPVs. The HBV SVPs are composed of large and small surface antigens (Jiang et al., 2015). Most of the viral particles are incomplete spherical and filamentous-like particles either containing viral genome (single stranded DNA, RNA, and RNA–DNA hybrids) or empty capsids (Hu and Liu, 2017). It is important to mention that approximately one of 10,000 viral particles is infectious. The SVPs are involved in disease progression and immune modulation in chronic carriers. Importantly, HBsAg carriers in cHBV infection have 25–37% increased risk for developing HCC (Kondo et al., 2013). Additionally, serum HBsAg level is correlated with intrahepatic HBV replication and transcription in HBeAg negative cHBV carriers (Li J. et al., 2017; Pfefferkorn et al., 2017). Therefore, it is believed that seroconversion of HBsAg in chronic infection can be a promising approach for cHBV treatment (McCaffrey et al., 2003).

Although the current FDA approved HBV treatments such as Lamivudine, Adefovir dipivoxil, Entecavir, Telbivudine, and IFN-α improve the anti-viral immune responses, they have a short-lasting, toxic, and high-cost limitations. Moreover, emerging of drug resistance strains is a major problem of the current drug regimen. Additionally, there are several reports of pre-existing of drug-resistant conferring substitutions in HBV polymerase (Han et al., 2009; Liu et al., 2010; Gomes-Gouvêa et al., 2015). In order to decrease the adverse effects of current HBV treatment, novel therapeutics have focused on stimulating host immune responses (Testoni et al., 2017; Cai et al., 2018; Klumpp et al., 2018). In this regard, preclinical try-outs of TLR-7 agonist, Vesatolimod (GS-9620), has shown promising interferon-dependent anti-HBV effect (Li L. et al., 2017; Niu et al., 2017; Boni et al., 2018). In a phase II, double-blind, randomized, placebo (PBO)-controlled study of the drug on cHB patients, it was shown that the GS-9620 was safe and well-tolerated in patients with cHB (Janssen et al., 2018). However, a functional cure will obtain trough HBsAg seroconversion. Current anti-HBV agents reduce viral replication and do not affect HBsAg levels in blood (Lindh et al., 2018). This may also restore the irresponsiveness of anti-HBV immune responses induced by HBsAg-bearing SVPs (Schluep et al., 2017). Here, we discussed various potent compounds targeting HBsAg, their actions, and advantages or disadvantages, which can be used in combination with other therapeutics in the future (**Table 1**).

**Table 1:** HBsAg inhibitors, their targets, and mechanism(s) of action.

Name	Target	Mechanism	cccDNA existence	Viral replicative intermediate	HBsAg secretion^∗^	Research phase	References
**Natural products**							
Osthole	HBsAg	HBsAg glycosylation	NA^∗∗^	NA	No	Nonclinical	Huang et al., 1996
*Cler. inerme*	HBsAg	Unknown	No	NA	No	Nonclinical	Mehdi et al., 1997
*Clem. sinensis*	HBsAg	Unknown	No	NA	No	Nonclinical	Mehdi et al., 1997
OjF	HBV DNA polymerase	Hypothetical inhibition of viral polymerase	NA	Reduced	No	Nonclinical	Wang et al., 2005
Hyperoside	Cellular targets	Unknown	NA	Reduced	No	Nonclinical	Wu et al., 2007
Alison A derivates	HBV DNA polymerase	Hypothetical inhibition of viral polymerase	NA	NA	No	Nonclinical	Zhang et al., 2008
PEI	Cellular factors	Unknown	NA	NA	No	Nonclinical	Huang et al., 2013
Luteolin-7-*O*-glucoside	Cellular factors	Reducing ROS release	NA	Suppressed	No	Nonclinical	Cui et al., 2017
**Small molecules**							
HBF-0259	Cellular HBsAg secretory factor	Hypothetical cellular secretory factors and glycosylation machine	Yes	Yes	No	Nonclinical	Dougherty et al., 2007; Mohebbi et al., 2016
BM601	Unknown	Interfering HBsAg aggregation in trans-Golgi apparatus	Yes	Yes	No	Nonclinical	Xu et al., 2014
Nicotinamide	SIRT1	HBV promoters modulation, HDAC inhibitor	NA	No	No	Nonclinical	Li et al., 2016
NJK14047	p38 MAPK	P38 MAPK inhibition	Reduced	No	No	Nonclinical	Kim et al., 2017
**Interfering RNAs**							
S-targeted siRNA	Viral S-coding transcripts	RNA interference	NA	No	No	Nonclinical	Klein et al., 2003
HBVU6.2	Viral S-coding transcripts	RNA interference	No	No	No	Nonclinical	McCaffrey et al., 2003
pGE-HPV2	HBV DNA polymerase	Hypothetical inhibition of viral polymerase	Yes	No	No	Nonclinical	Liu et al., 2005
ARC-520	All viral transcripts	RNA interference	NA	No	No	Phase I/II	Lanford et al., 2013; Yuen et al., 2014; Schluep et al., 2017
**Other types of HBsAg inhibitors**						
Mycophenolic acid	Monophosphate dehydrogenase	HBV polymerase suppression through guanosine depletion	Undetectable	Reduced	No	Nonclinical	Gong et al., 1999
C4D2-BsAb	HBsAg	Binding to and induce conformational changes within HBsAg	NA	NA	No	Nonclinical	Tan et al., 2013
HNF1α	NF-κB p65 and LHBsAg	NF-κB activation	NA	No	No	Nonclinical	Lin et al., 2017

*^∗^No one of inhibitors have reported completely and long-lasting suppression of HBsAg. ^∗∗^Based on the mechanism of action of the inhibitor, it is not applicable or not reported in the study.*

## Different Classes of HBsAg Inhibitors

### Natural Products

Since conventional chemical drugs’ adverse effects and drug resistance are known, natural products and related bioactive compounds have gained increasing attention from physicians and medical researchers. Natural products as a class of traditional medicine are sophisticated and time-honored form of healthcare in many countries.

In Chinese medical herb, Osthole extracted from *Angelica pubescens* Maxim (Du Huo) root has screened for HBsAg secretion inhibition (Huang et al., 1996). In MS-G2 and Huh-7 cells, Osthole concentrations from 5 to 20 μg/mL have shown remarkable HBsAg secretion inhibition from 23.5 to 60.5% and 18.4 to 70.1% after days 3–6, respectively (Huang et al., 1996). Results of the study on Osthole suggest specific post-transcriptional inhibition of both small and middle HBsAg secretion^8^. The study revealed that hyper-glycosylation of HBsAg is a mechanism underlying Osthole action as a potent inhibitor of HBsAg (Huang et al., 1996).

Terrestrial plants’ extracts have been evaluated for their anti-HBV activities (Mehdi et al., 1997). Of 19 terrestrial plants’ extracts from different parts of the herb, some have shown more than 50% HBsAg secretion inhibition. Of which, two extracts from *Clerodendrum inerme* (family Verbenaceae) with 85% and *Clematis sinensis* (family Ranunculaceae) with almost 60% HBsAg inhibition were reported to be strongest with no cytotoxicity in HepG2.2.15 cells (Mehdi et al., 1997). While the compounds have suppressed HBV replication, the extracts had no long-lasting inhibitory effects on HBsAg secretion.

The active compound of OjF has been used widely for the treatment of a broad range of symptomatic human diseases such as HBV infection (Wang et al., 2005). The OjF has suppressed HBsAg secretion in a dose- and time-dependent manner. There were no remarkable side effects in HBV-infected ducklings treated with OjF as well (Wang et al., 2005). The hypothetical mechanism of OjF suggests that it may prevent HBV DNA polymerase activity and subsequently HBsAg transcription and production (Wang et al., 2005). While animals’ treatment with > 50 g/L of OjF has shown extended HBV reproduction suppression, 3 days after OjF cessation, HBV replication restored. Interestingly, viremia has also decreased as high as 64% following OjF treatment in animal models.

Hyperoside (quercetin-3-*O*-galactoside), a flower extract of *Abelmoschus manihot* (L) medik, is another natural product with anti-HBV activity (Wu et al., 2007). This edible herb has a broad range of medication usage. The HBsAg producing cell line, HepG2.2.15, and infected ducklings were used for investigating anti-HBV activity of hyperoside (Wu et al., 2007). The HBsAg secretion inhibition rates were 82.27% at the dose of 5 mg/L. Non-toxic dose of hyperoside has shown liver histopathological improvements indicating reduced levels of viremia (Wu et al., 2007). However, the mechanism of action of hyperoside-induced HBsAg production suppression is unknown.

The natural product Alisol A’s is a protestane-type triterpens. Hydroxyl (OH)-substituted Alisol A lead compounds have anti-HBsAg activities (Zhang et al., 2008). Future hit-to-lead optimization of protestane-type triterpene compounds would be a promising anti-HBV achievement. Like other FDA-approved HBV polymerase inhibitors, Alisol A derivates might have an identical mechanism of action (Zhang et al., 2008). However, further analysis is needed to understand its exact role in HBsAg secretion inhibition. Accordingly, neither viral DNA synthesis nor production of HBV transcripts had not been investigated.

There are further studies, which have been worked on the natural products against HBV infection. A basic, thermo-stable, polypeptide PEI extracted from Jelly Fig Achenes (Ficus awkeotsang Makino) has been studied for its anti-HBsAg expression potential (Huang et al., 2013). In that study, two cell lines bearing endogenous and integrated forms of HBV genome, Hep-3B and Huh-7, have been used for assessing HBsAg expression in the PEI treated supernatants. In average, 80% decreased of HBsAg gene expression had observed *in vitro*. Meanwhile, there was a decreased in the antigen secretion inhibition in Hep-3B containing endogenous form of the virus genome (Huang et al., 2013). The main biological function of PEI is known to induce programmed cell death through the intrinsic caspase-dependent pathway. The mechanism of action underlying HBsAg secretion inhibition in supernatants under PEI treatment is unknown. Furthermore, analysis of the different viral processes, including DNA replication, RNA transcription, and viral/SVP production, has also needed to be investigated (Huang et al., 2013).

Active compound, Luteolin-7-*O*-glucoside, extracted from leafy vegetable lettuce (*Latuca satica L.*) has been shown to significantly reduce HBsAg production (>82%; Cui et al., 2017). The plant extract had no cellular toxicities at lower concentrations. The viral RNA is a biomarker for activity of HBV transcription (Cui et al., 2017). Accordingly, the viral RNA was significantly reduced (43.9%), which was consistent with reduced HBV replication (Cui et al., 2017). Inoculation of Luteolin-7-*O*-glucoside along with 3TC or IFN-α has been shown to have synergistic anti-HBsAg production effects. However, it was not significantly differing from that of the extract alone (Cui et al., 2017). HBsAg secretion inhibition mechanism of Luteolin underlies beneath the ability of the compound to inhibit mitochondrial production of ROS (Cui et al., 2017), indicating the role of ROS in HBV lifecycle.

Discoveries of anti-viral natural products are still an expanding approach. The studies clearly indicate a potential utility of traditional herbal medicine in cHBV treatment, especially in HBsAg inhibition. Further studies are needed to improve and specify their potencies and uncover mechanisms of the products’ activities. In this regard, studies in animal models would be helpful.

### Small Molecules

Drug discovery approach is a promising strategy for finding novel anti-viral inhibitors (Lee et al., 2011). High-throughput screening of four widespread small molecule libraries composed of over 80,000 drug-like leads, led to identification of potent anti-HBV compound, HBF-0259 (Dougherty et al., 2007). In a time- and dose-dependent manner, HBF-0259 specifically targets all forms of HBsAg in HBsAg expressing cells, HepH2.2.15 and HepDE19 (Dougherty et al., 2007). This mechanism of action may be underlain beneath of its interaction with cellular secretory or HBsAg post-translationally modifying factors (Dougherty et al., 2007; Mohebbi et al., 2016). Long-term treatment assay of HBF-0259 has been shown no traceable level of glycosylated forms of HBsAg in the supernatant (Dougherty et al., 2007). Meanwhile, only intracellular non-glycosylated forms of HBsAg have been observed. This indicates that HBF-0259 may target cellular counterparts involved in HBsAg modification and secretion (Mohebbi et al., 2016). Moreover, HBF-0259 had no significant effect on HBV DNA replication.

Benzimidazole and its derivatives have therapeutic potency, including anti-microbial, anti-viral, anti-tumoral, and anti-allergic effects (Walia et al., 2011; El Rashedy and Aboul-Enein, 2013). BM601 is a Benzimidazole derivative with an ability to inhibit HBsAg secretion (Xu et al., 2014). BM601 inhibits HBsAg secretion and subsequently HBV viral particle formation *in vitro*. HBV DNA replication, transcription, and nucleocapsid synthesis were not altered following BM601 treatment in HepG2.2.15 cell line. Also, it does not change cellular protein synthesis, ROS stimulation, and ER stress (Xu et al., 2014). Importantly, BM601 induces HBsAg secretion inhibition and subsequently HBV viral particle formation through alteration of HBsAg displacement with high cytoplasmic distributions, but it does not alter HBsAg glycosylation patterns (Xu et al., 2014).

Nicotinamide is the active form of nicotinic acid, niacin (vitamin B3), and a precursor of NAM adenine dinucleotide (NAD^+^). NAM is the most potent inhibitor of SIRT1 with known anti-viral effects (Koyuncu et al., 2014). Different concentrations of NAM have been used for evaluating anti-HBV activity in both HepG2.2.15 and HepAD38 cells as well as HBV transgenic mice model (Li et al., 2016). Furthermore, suppression of HBsAg expression was dose-dependent following NAM treatments. Highest inhibition of HBsAg expression was at 16 mM NAM concentration with production of no viral particles *in vitro* (Li et al., 2016). Luciferase reporter assay has been indicated direct interaction of NAM with all four HBV, SpI, SpII, core, and X promoters (Li et al., 2016). Indirectly, NAM was found to suppress HBV transcription and replication by reduction in cellular transcription factors such as the AP-1, C/EBPa, and PPARα levels (Li et al., 2016).

Biphenylamid non-nucleoside derivate, NJK14047, is active in inhibition of p38 MAPK (Kim et al., 2017). Accordingly, NJK14047 has been shown the greatest p38 MAPK inhibitory effect highly correlated (85%) with HBsAg secretion inhibition. HBsAg secretion has shown to be inhibited in both HBV integrated and transient transfected HepG2 cell lines as high as 70% and 90%, respectively. Similar HBsAg secretion inhibition has been also observed in a cell line infected with HBV expressing NTCP after NJK14047 treatment (Kim et al., 2017). Higher dose of NJK14047 small molecule was needed for obtaining sufficient HBV viral DNA, mRNA, and viral particle production (Kim et al., 2017). The novelty of the study was to target cellular factors involved in HBV infection.

Cyclophilin A inhibitors are known to have anti-viral properties (Dawar et al., 2017). In this regard, Shimura et al. (2017) have been shown potential of Cyclosporin (Cs) derivates to inhibit HBV entry. In that study, which was conducted to produce anti-HBV agents to inhibit HBV, it was observed that Cs derivates are potent to prevent viral entry through NTCP pathway (Shimura et al., 2017). Effects of the derivates (SCY450 and SCY995) on HBV entry were evaluated in cell line HepG2-hNTCP-C4 over-expressing NTCP. The compounds were not immunosuppressant. They also had no effect on HBV replication. In a dose- and time-dependent manner, the HBsAg level was significantly decreased. The advantage of the derivates was in their role to target NTCP without affecting its main function, bile acid transportation. The compounds have also been potentiated to prevent entry of several HBV genotypes (A, B, C, and D). In addition, the derivates could inhibit HBV variant containing three revers-transcriptase substitutions conferring entecavir resistance (Shimura et al., 2017).

Considering that small molecules inhibit HBV infection and HBsAg secretion, further optimization may provide clinically applicable therapies. In this regard, discoveries of small molecules targeting known cellular factors involved in HBV pathogenesis or the virus itself are much target-specific and easy-to-obtain procedure. Optimization of this potential anti-HBV drug-like compound would be promising in the future of HBV therapy.

### RNA Interference

There is accumulating data on the RNA interference utility in HBV treatment. HBsAg targeted siRNAs have been evaluated in a novel NMRI animal model of HBV infection in order to suppress the antigen production (Klein et al., 2003). Results of the study have shown a significant decrease of HBsAg and HBeAg expression. As an important therapeutic benefit of using siRNA is that the siRNAs are well constructed to target the virus regulatory elements. However, short-lasting effect of the presented siRNA (11 days) is a disadvantage, which may be resulted from inefficient transfection of mice hepatocytes (10%; Klein et al., 2003).

HBV replication supportive Huh-7 cells and two immunocompetent C57BL/6 and immunocompromised NOD SCID mice models transfected with HBV pTHBV2 clone have been used for investigation of anti-HBV effect of shRNAs (McCaffrey et al., 2003). In that study, four potent double-stranded shRNAs have been designed to target HBsAg transcription (HBVU6no.1–4). Of those, only HBVU6no.2 showed significant HBsAg suppression *in vitro* (94 ± 0.59%) in a time-dependent manner. In NOD mice with no interfering immunity, treatment with HBVU2no.2 has reduced HBsAg level more than 85% in 1 week. The results were accompanied with no observation of cccDNA in transfected mice and no evidence of detectable levels of HBV replicative intermediates (McCaffrey et al., 2003). This report presents a rational evidence of anti-HBV approach through targeting the viral transcripts and HBV suppression in both *in vivo* and *in vitro*.

In the last decade of the cHBV infection therapy, ARC-520 RNA interference therapy is promising because of accelerating HBsAg seroclearance and host immune reconstitution (Yuen et al., 2014). Single intravenous injection (2 mg/kg) of the siRNA in HBV mice models showed a good reduction in HBV viral load and HBsAg secretion with no toxicity (Lanford et al., 2013). Moreover, in chronically infected adult non-human primates, HBsAg decreased up to 96% with the same dose and a second boost of ARC-520 (3 mg/kg). This result was promising in big-sized cHBV animal models. However, after 30 days of first injection, HBsAg levels have rescaled, partly due to short half-life of ARC-520 siRNAs components (Lanford et al., 2013; Schluep et al., 2017). This disadvantage may come along with the fact that by increasing body weight, the dose of the siRNA should be heightened, which may cause toxicity. This concern has been answered in phase I clinical study of ARC-520, where hypersensitivity reaction (resolved by oral antihistamine administration) and no further cytotoxicity were observed in 36 healthy volunteers (Schluep et al., 2017). Further adverse effects were transient or just because of subject’s pre-dose issues. In the next double-blinded, PBO-controlled phase IIa study, single intravenous administration of 1 mg/kg, 2 g/kg, and 3 mg/kg of ARC-520 were done to the three cohorts of chronically HBV-infected adult patients, respectively (Yuen et al., 2014). In that study, 31%–51% HBsAg declines were observed in cohort 1 and 2 on day 85, respectively, and the cohort 3 is still blinded (Yuen et al., 2014).

In a study, authors have used a 29-base sequence stretch of HepG2.2.15 genome for the design of siRNA (pGE-HPV2) targeting HBV genome (Liu et al., 2005). pGE-HPV2 was potent with HBsAg secretion inhibition rate of 80% in a time-dependent manner (50% after 24 h post-transfection; Liu et al., 2005). The pGE-HPV2 is designed to target HBV DNA polymerase. This molecule may prevent viral replicase production and subsequently decrease HBsAg gene transcription. Further information from this study is not known. However, it could be concluded that the siRNA may interfere with viral replication.

Employment of siRNAs in HBV infection inhibition has been considered as a promising approach. This is supported by two clinical trial phases of ARC-520. The advantage of ARC-520 over other mentioned siRNAs is that ARC-520 targets all forms of HBV transcripts, and it is well designed to localize into hepatocytes efficiently with minimized cytotoxicity. The most important disadvantage of RNA interference may be the incidence of scape mutant strains. However, ARC-520 with formulation of two multisite-targeting siRNAs has broad-range HBV genotype coverage and reduces the chance of scape mutant emergence. This might ultimately lead to clinical therapy for HBV infection.

### Other Classes of HBsAg Secretion Inhibitors

There are also other types of HBsAg secretion inhibitors. Immunosuppressive Mycophenolate (MMF) active compound, MPA, is a monophosphate dehydrogenase (MP-DH) inhibitor. It can inhibit HBsAg secretion in HepG2.2.15 cell line at low concentrations (Gong et al., 1999). The mechanism of HBsAg secretion inhibition by MPA underlies its potential to depleting cellular exogenous guanosine source (GTP) and subsequently viral DNA polymerase inactivation (Gong et al., 1999).

Two mAbs C4G4 and D2H2 have been demonstrated to target two circularized epitopes within the HBsAg loop domain (Tan et al., 2013). A bispecific antibody, C4D2-BsAb, engineered from C4G4 and D2H2 mAbs has been shown to recognize two HBsAg loop domain epitopes in one time. This prevents interaction of HBV with its cellular surface receptor(s). Accordingly, pre-treatment of HepRG cells and treatment of cell line producing HBsAg, PLC/PRF/5, have shown remarked decrease of HBsAg secretion (Tan et al., 2013). C4D2-BsAb targets circularized epitopes within HBsAg loop domain, and it can be used for prevention therapy approaches (Tan et al., 2013). However, investigation of infected chimpanzees revealed short (few days) lasting of HBsAg suppression with antibodies (Heijtink et al., 1999). The C4D2-BsAb action may be through induction of conformational changes as well as conflicting with virus–receptors interactions.

Other immune modulators have also shown to have anti-HBV activity. Accordingly, HNF1α, an LETF, can downregulate HBV gene expression and replication (Lin et al., 2017). Indirect effect of HNF1α in reduction of HBsAg secretion and viral particle production is through upregulation of p65, a nuclear marker for NF-kB activation, and downregulation of p65 inhibitor HDAC2, *in vitro* (Lin et al., 2017). Viral infection activates NF-kB signaling, which leads to nuclear accumulation of NF-kB and downstream gene transcription, triggering cellular events like apoptosis (Su and Schneider, 1996; Purcell et al., 2001). It can be concluded that apoptosis induced by HNF1α could be a hypothetical mechanism underlying HBsAg secretion inhibition from infected cells.

## Conclusion and Future Perspectives

According to the meeting of American Association for the Study of Liver Diseases and the European Association for the Study of the Liver (Lok et al., 2017b), cHBV cure defines as loss of HBsAg in serum, eradication of integrated viral DNA and residual cccDNA, and resolution of damaged hepatocytes with decreased risk of HCC (Lok et al., 2017b). The infected hepatocytes of patients with cHBV are reservoirs for active/inactive cccDNAs and integrated form of viral double-stranded linear DNA, which both produce HBsAg and subsequently SVPs (Allweiss and Dandri, 2017; Huang et al., 2018). It seems that using novel potent and non-toxic HBsAg inhibitors might restore exhausted immune response, which induced by SVPs (Schluep et al., 2017).

Further obstacles beneath HBV infection treatment underlie in frequent drug resistance mutants. There are several reports regarding to drug resistance mutants in treatment-naïve patients (Han et al., 2009; Pastor et al., 2009; Solmone et al., 2009; Tenney et al., 2009; Liu et al., 2010; Sayan et al., 2010; Salpini et al., 2011; Vutien et al., 2014; Gomes-Gouvêa et al., 2015). This brings burden of screening, defining new anti-viral regimen, and more importantly transmission chance of mutant strains. Using novel non-nucleos/tide therapeutics may improve the need of mutants screening. However, the rise of scape mutants after siRNAs therapy may still be a concern (McCaffrey et al., 2003).

For achieving to the ideal of HBsAg clearance, sustained HBsAg secreting cell lines and animal models have been used. Meanwhile, HBsAg secretion inhibition levels are always significantly lower in cell lines with integrated viral genome than that in other transiently transfected hepatoma cells (Kim et al., 2017). This is because of constant expression of HBsAg, which is independent of virus itself. It also implies the importance of HBsAg secretion inhibition in such models for evaluating the efficiency of novel HBV therapeutics. One other limitation for the study of HBV pathogenesis is the unavailability of inexpensive, easy-to-handle, small animal models (Klein et al., 2003). Therefore, there are some advances in development of such animals (Klein et al., 2003; Wang et al., 2005; Li et al., 2016). This provides better insight of HBV infection and therapeutics approaches. Animal models like C57BL/6 and NMRI mice, hatched ducklings, and chimpanzees are used for anti-HBV therapeutics. However, big-size chronically infected chimpanzees are suitable animal models for development and evaluation of novel potent anti-HBV agents (Wang et al., 2005; Zhang, 2015).

**FIGURE 1:**
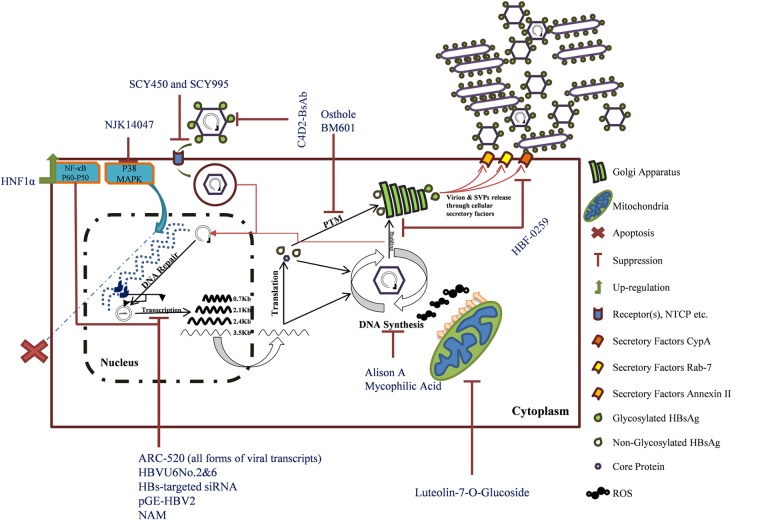
The mechanisms of HBsAg inhibitors’ action. Hepatitis B virus life cycle is illustrated. Inhibitors are named in blue. The integrated form of HBV is shown as dark blue within host genome (blue dashed line). The bold viral transcripts are those that are also transcribed from the integrated viral genome (Tu et al., 2017).

In this review, HBsAg inhibitors were clustered into small molecules, natural products, interfering RNAs, and another group contained immunological therapeutics. Based on the mechanism of action, some factors are targeting viral components, including siRNAs, shRNAs, HBF-0259, MPA, pGE-HPV2, NAM, C4D2-BsAb, OjF, and Alisol A. Further inhibitors, including Osthole, BM601, NJK14047, Luteolin-7-*O*-glucoside, SCY450 and SCY995, and HNF1α, all have cellular targets. Five other inhibitors’ (PEI, *Cler. inerme*, *Clem. sinensis*, BM601, and hyperoside) mechanisms of action are still unknown (**Figure 1**). There is a novel HBV receptor inhibitor, Myrcludex B, that targets NTCP but it has no effect of HBsAg secretion (Volz et al., 2013; Blank et al., 2018). The compound is in pre-clinical trial and shows promising results (Blank et al., 2016). The discussed compounds are not all introduced to human trial, and some may never rich in. This is because of some critical checkpoints at which the virus activities should be evaluated: (1) decreasing HBsAg level must be correlated to reduction of serum HBV pgRNA levels, implicating downregulated residual cccDNA activities and (2) restoration of cellular immunity targeting infected hepatocytes containing different forms of HBV genome (Lok et al., 2017a; Cornberg and Manns, 2018).

HBsAg inhibitors are discussed in detail (**Figure 1** and **Table 1**). In addition, there is this believe that host immune restoration followed by HBsAg seroconvergence would be the functional HBV cure. Therefore, the present study did not attend much about other aspects of the virus pathogenesis factors like HBeAg and HBcAg. Furthermore, different classes of therapeutic anti-cHBV vaccine in human trials are reviewed in (Ghasemi et al., 2016).

In conclusion, small molecules and natural products are well-tolerated anti-HBsAg candidates, which if possible might provide a better insight on HBV–cell counterpart interactions and pathogenesis in the future. As reviewed here, no HBsAg inhibitors have been enabled to suppress secretion of the antigen completely. This might be due to residual cccDNA and or integrated viral genome. Therefore, a “complete functional cure” will achieve through both suppression of HBsAg and elimination of infected hepatocytes. Because of genomic nature of HBV, interfering RNAs by targeting overlap regions of HBV genome will have promising results. Further therapeutics like anti-HBsAg aptamers neutralizing viral particles (Xi et al., 2015), HBV receptor inhibitors (Kaneko et al., 2015; Wang et al., 2015; Shimura et al., 2017), and CRISPR-Cas9 (Liu et al., 2018; Moyo et al., 2018; Yang and Chen, 2018) targeting integrated and cccDNA genome would be the ideal therapies in the future.

## Author Contributions

AM contributed to the design and concept of the paper, literature review, illustration, sorting data, and drafting the article. NL performed the literature review and data collection. NK performed the literature review and classification of HBsAg inhibitors. ATah performed the critical review, revisions, and consulting for improving the manuscript. ATah performed the critical review and revisions and guided the study.

## Conflict of Interest Statement

The authors declare that the research was conducted in the absence of any commercial or financial relationships that could be construed as a potential conflict of interest.

## Abbreviations

AP-1: activator protein 1
cccDNA: covalently closed circular DNA
C/EBPa: CCAAT/enhancer-binding protein α
cHBV: chronic HBV
CypA: cyclophilin A
CRISPR/Cas: clustered regularly interspaced short palindromic repeats
ER: endoplasmic reticulum
HBsAg: HBV surface antigen
HBV: hepatitis B virus
HCC: hepatocellular carcinoma
HDAC2: histone deacetylase 2
HNF1α: hepatocyte nuclear factor 1α
IFN-α: interferon-α
LETF: liver-enriched transcription factor
mAbs: monoclonal antibodies
MAPK: mitogen-activated protein kinase
MPA: mycophenolic acid
NAM: nicotinamide
NTCP: Na+-taurocholate co-transporting polypeptide
OjF: *Oenanthe javanica* flavones
PEI: pectinesterase inhibitor
PPARα: peroxisome proliferator-activated receptor α
ROS: reactive oxygen species
shRNAs: short hairpin RNAs
siRNAs: small interfering RNAs
SIRT1: sirtuin 1
SPVs: sub-viral particles
TLR-7: toll-like receptor 7.
